# Bacterial Community Dynamics in Dichloromethane-Contaminated Groundwater Undergoing Natural Attenuation

**DOI:** 10.3389/fmicb.2017.02300

**Published:** 2017-11-22

**Authors:** Justin Wright, Veronica Kirchner, William Bernard, Nikea Ulrich, Christopher McLimans, Maria F. Campa, Terry Hazen, Tamzen Macbeth, David Marabello, Jacob McDermott, Rachel Mackelprang, Kimberly Roth, Regina Lamendella

**Affiliations:** ^1^Lamendella Laboratory, Juniata College, Department of Biology, Huntingdon, PA, United States; ^2^Wright Labs, LLC, Huntingdon, PA, United States; ^3^Bredesen Center for Interdisciplinary Research and Graduate Education, University of Tennessee, Knoxville, TN, United States; ^4^Biosciences Division, Oak Ridge National Laboratory (DOE), Oak Ridge, TN, United States; ^5^Institute for a Secure and Sustainable Environment, University of Tennessee, Knoxville, TN, United States; ^6^Department of Microbiology, University of Tennessee, Knoxville, TN, United States; ^7^Department of Civil and Environmental Engineering, University of Tennessee, Knoxville, TN, United States; ^8^Department of Earth and Planetary Sciences, University of Tennessee, Knoxville, TN, United States; ^9^CDM Smith, Edison, NJ, United States; ^10^Department of Biology, California State University Northridge, Northridge, PA, United States

**Keywords:** dichloromethane, DCM, 16S rRNA, biodegradation, xenobiotic, groundwater, monitored natural attenuation, bacterial community

## Abstract

The uncontrolled release of the industrial solvent methylene chloride, also known as dichloromethane (DCM), has resulted in widespread groundwater contamination in the United States. Here we investigate the role of groundwater bacterial communities in the natural attenuation of DCM at an undisclosed manufacturing site in New Jersey. This study investigates the bacterial community structure of groundwater samples differentially contaminated with DCM to better understand the biodegradation potential of these autochthonous bacterial communities. Bacterial community analysis was completed using high-throughput sequencing of the 16S rRNA gene of groundwater samples (*n* = 26) with DCM contamination ranging from 0.89 to 9,800,000 μg/L. Significant DCM concentration-driven shifts in overall bacterial community structure were identified between samples, including an increase in the abundance of Firmicutes within the most contaminated samples. Across all samples, a total of 6,134 unique operational taxonomic units (OTUs) were identified, with 16 taxa having strong correlations with increased DCM concentration. Putative DCM degraders such as *Pseudomonas*, *Dehalobacterium* and *Desulfovibrio* were present within groundwater across all levels of DCM contamination. Interestingly, each of these taxa dominated specific DCM contamination ranges respectively. Potential DCM degrading lineages yet to be cited specifically as a DCM degrading organisms, such as the *Desulfosporosinus*, thrived within the most heavily contaminated groundwater samples. Co-occurrence network analysis revealed aerobic and anaerobic bacterial taxa with DCM-degrading potential were present at the study site. Our 16S rRNA gene survey serves as the first *in situ* bacterial community assessment of contaminated groundwater harboring DCM concentrations ranging over seven orders of magnitude. Diversity analyses revealed known as well as potentially novel DCM degrading taxa within defined DCM concentration ranges, indicating niche-specific responses of these autochthonous populations. Altogether, our findings suggest that monitored natural attenuation is an appropriate remediation strategy for DCM contamination, and that high-throughput sequencing technologies are a robust method for assessing the potential role of biodegrading bacterial assemblages in the apparent reduction of DCM concentrations in environmental scenarios.

## Introduction

Uncontrolled spills of dichloromethane, or DCM, have been documented to cause widespread groundwater contamination across the United States (Robinson et al., 2009). Due to its carcinogenic and potentially lethal effects in humans (Program, 1986; Miligi et al., 2006; EPA, 2011; Gold et al., 2011; Schlosser et al., 2015) the US Environmental Protection Agency has set stringent maximum contamination limit of DCM at 5 parts per billion (μg/L) in public or private well water sources (EPA, 2011). The DCM is a dense non-aqueous phase liquid (DNAPL) that migrates vertically through the unsaturated and the saturated groundwater zone because of its high relative density. The DNAPLs can form discontinuous globules or ganglia due to capillary retention within the pore space of soils or bedrock as they migrate vertically (Pankow and Cherry, 1996) and can form pools of high-saturation DNAPL above layers or lenses of lower-permeability soil and rock media (Seagren et al., 1993; Gerhard and Kueper, 2003). Sites containing DNAPL mass are long-term sources of groundwater contamination. Bedrock aquifers contaminated with DNAPLs are particularly challenging to clean up due to complexity of the fracture system through which DNAPL migrates. In addition, high concentrations of DCM can diffuse into porous bedrock matrix creating significant secondary sources of contamination (Chapman and Parker, 2005). As a result, technology options for cleanup of DNAPL aquifer systems are often costly and impractical. Therefore, evaluation of the intrinsic capacity of an aquifer to naturally attenuate DCM is important to ensure that cleanup decisions are providing clear benefits and are sustainable.

One key element in understanding the aquifer attenuation capacity is consideration of the factors that influence movement of contaminants. Identifying microbial populations capable of DCM degradation throughout the contaminant plume can support a fate and transport evaluation and confirm that declining trends in concentration are a result of degradation, and not physical processes such as dilution and/or matrix diffusion.

Biodegradation of DCM by a variety of bacterial and eukaryotic species has been known for more than three decades (Brunner et al., 1980; Ravi et al., 2013). DCM can be utilized as a sole carbon and energy source under both aerobic and anaerobic conditions by several organisms within contaminated environments (Krausova et al., 2003; MacDonald and Gordon, 2005). The majority of the known DCM-degraders are aerobic methylotrophic bacteria, including strains within the *Methylobacterium* (Gälli and Leisinger, 1985; Vuilleumier et al., 2009; Firsova et al., 2010) and *Hyphomicrobium* (Kohler-Staub and Leisinger, 1985; Diks and Ottengraf, 1991; Urakami et al., 1995; Nikolausz et al., 2005; Vuilleumier et al., 2011) genera. Aerobic degradation of DCM by these consortia is catalyzed by the enzyme DCM dehalogenase, which converts DCM into two molecules of HCl and formaldehyde (Brunner et al., 1980; La Roche and Leisinger, 1990). While most DCM-degraders are aerobic, some bacteria are able to degrade it anaerobically, in which DCM is transformed to methane, carbon dioxide, and acetate (Freedman and Gossett, 1991; Stromeyer et al., 1991; Braus-Stromeyer et al., 1993; Meßmer et al., 1996; Kaufmann et al., 1998; Mägli et al., 1998; De Best et al., 2000; Kleindienst et al., 2016). Bioreactor studies performed using DCM-degrading strains demonstrate that degradation rates are higher under aerobic conditions compared to anaerobic degradation (Stucki, 1990; De Best et al., 2000). Currently, the literature of DCM-degrading organisms has focused on microcosm and bioreactor studies of cultivated organisms. While these studies provide insights into the identity of some DCM-degraders and molecular mechanisms involved in DCM degradation, they do not provide a complete view of the diversity and functional capacity of microbial communities within contaminated environments.

In our study, we sought to investigate bacterial community responses to DCM within groundwater, and identify distinct assemblages of bacteria capable of thriving in DCM-rich environments. Bacterial community structure was assessed within a groundwater DCM DNAPL source and dissolved phase plume from an undisclosed manufacturing facility in New Jersey. 16S rRNA analysis was conducted on 26 groundwater samples collected from wells with levels of contamination ranging from 0.89 to 9,800,000 μg/L. We found that bacterial community structure shifted across DCM contamination level such that increasing DCM contamination enriched unique DCM-degrading assemblages within these groundwater environments. This represents the first study to leverage a high-throughput sequencing technology to assess the comprehensive bacterial community response to varying concentrations of DCM in groundwater environments.

## Materials and Methods

### Site Description

**FIGURE 1:**
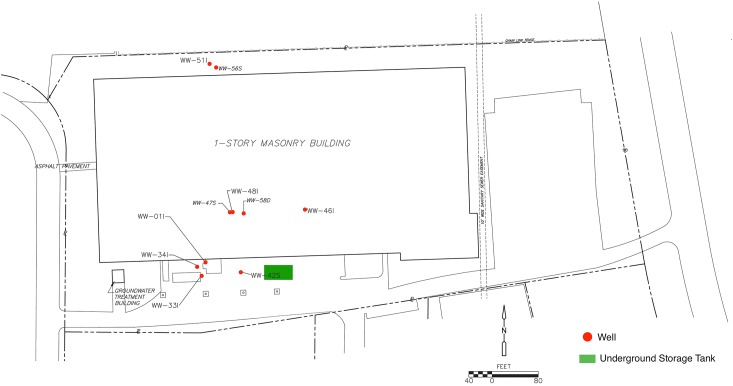
A sitemap of the contamination zone was generated in Inkscape (version 0.91) by editing a CADD base-map used in groundwater sampling report submittals. Additional monitoring wells not included in 16S rRNA analysis as well as site location details were removed from the map. Site investigation data suggest that the DCM spill originated from the former Underground Storage Tank (UST) area (highlighted in green) and migrated downward and to the northwest, primarily controlled by joint and bedding plane fracture permeability, consequently increasing DCM concentration within wells 47S, 01I, 33I, and 34I.

An underground storage tank was used for DCM and discharged an unknown quantity of DNAPL into the subsurface prior to 1986. Although the former tanks and contaminated soils were removed in 1989 and 1990, DCM-contaminated groundwater in the bedrock aquifer remain at depth with concentrations detected greater than 10,000,000 parts per billion (μg/L). A groundwater pump and treat system was operated from 1995 to 2009 as the original cleanup plan, but dissolved concentrations remained orders of magnitude above cleanup goal of 5 μg/L. In addition, the system was not removing significant amounts of DCM mass and the data indicated that the pump and treat system did not have an appreciable effect on the plume extent. The system was shut off permanently in 2009 and a study was initiated to evaluate the attenuation capacity of the aquifer and determine a revised remedial strategy. While high contaminant levels persist, overall concentrations in the source area have been relatively stable, with a downward trend in concentrations in the down gradient plume indicating that the plume is stable and retracting. A site map generated within Inkscape (version 0.91) detailing the sampling well distribution as well as the origin of the DCM spill is presented in **Figure 1**.

### Geology/Hydrogeology

The site is underlain by unconsolidated weathered shale of the Passaic formation, above fractured shale bedrock, with bedrock as shallow as 10 feet below grade. The static depth to water in the bedrock aquifer is approximately 25 feet below the ground surface (bgs). The low conductivity of the bedrock onsite results in low yielding wells, typically 0.5–2.5 gallons per minute overall. Two laterally extensive water-bearing zones at depths of approximately 40 feet (referred as shallow “S” in the well designation) and 70 feet bgs (referred to as intermediate “I” in the well designation) across the site contain the majority of groundwater contamination. However, one deeper well, WW-58D, screened at a depth of 120–140 feet bgs has exhibited significant concentrations and so was included in this study.

### Sample Collection

Groundwater samples (*n* = 26) were collected quarterly starting in October of 2013 until August 2014 from 10 groundwater sampling wells surrounding the spill site. Wells chosen for bacterial community analysis were selected based on historical geochemical data to obtain a sample distribution among relatively high DCM contamination levels (47S, 33I, 01I, 34I), wells with moderate contamination (46I, 48I, 58D), and wells with low DCM concentrations (42S, 51I, 56S). **Table 1** provides a comprehensive summary of well identifications, sampling information and measured water chemical parameters within each collected sample.

**Table 1:** Measured water chemistry parameters and DCM concentrations within all sampled monitoring wells (*n* = 10) over the 4 sampling quarters.

	Water chemistry	Q4	Q1	Q2	Q3
		October, 2013 (99)	February, 2014 (100)	May, 2014 (101)	August, 2014 (102)
WW-01I	DCM (μg/L)	1.2	60,000	160,000	XXX
	pH (pH Units)	5.91	5.75	4.44	xxx
	Redox (mV)	21.4	-18.5	21.9	xxx
	DO (mg/L)	0.33	0.64	0.23	xxx
WW-33I	DCM (μg/L)	140,000	98,000	89,000	XXX
	pH (pH Units)	7.09	6.35	5.64	xxx
	Redox (mV)	-84.2	-59.9	-30.6	xxx
	DO (mg/L)	0.02	0.56	0.53	xxx
WW-34I	DCM (μg/L)	XXX	690	46,000	790,000
	pH (pH Units)	xxx	8.3	7.91	7.27
	Redox (mV)	xxx	-150.1	-50.9	-143.9
	DO (mg/L)	xxx	1.5	1.7	0.03 (0.23)
WW-42S	DCM (μg/L)	3.6	XXX	7	2.1
	pH (pH Units)	7.8	xxx	7.17	6.29
	Redox (mV)	3.9	xxx	7	78.8
	DO (mg/L)	0.45	xxx	2.47	0.26 (0.39)
WW-46I	DCM (μg/L)	XXX	0.89	77	5.9
	pH (pH Units)	xxx	7.97	8.11	7.03
	Redox (mV)	xxx	-70	2.4	-34.2
	DO (mg/L)	xxx	0.07	0.23	0.06 (0.15)
WW-47S	DCM (μg/L)	9,800,000	6,500,000	8,800,000	5,500,000
	pH (pH Units)	6.41	6.67	6.34	6.05
	Redox (mV)	88.6	283	47.2	-15.3
	DO (mg/L)	0.84	0.39	1.7	0.07 (0.15)
WW-48I	DCM (μg/L)	XXX	5.2	180	24
	pH (pH Units)	xxx	6.49	6.41	5.96
	Redox (mV)	xxx	137.6	7	43.1
	DO (mg/L)	xxx	0.38	0.41	0.05 (0.32)
WW-51I	DCM (μg/L)	XXX	3.4	XXX	XXX
	pH (pH Units)	xxx	7.39	xxx	xxx
	Redox (mV)	xxx	46.3	xxx	xxx
	DO (mg/L)	xxx	0.96	xxx	xxx
WW-56S	DCM (μg/L)	XXX	6.4	XXX	1.6
	pH (pH Units)	xxx	8.07	xxx	6.95
	Redox (mV)	xxx	139.2	xxx	105.5
	DO (mg/L)	xxx	7.7	xxx	7.55 (8.62)
WW-58D	DCM (μg/L)	XXX	73	XXX	XXX
	pH (pH Units)	xxx	8.4	xxx	xxx
	Redox (mV)	xxx	-113.6	xxx	xxx
	DO (mg/L)	xxx	0.14	xxx	xxx


*Samples without data listed did not yield detectable bacterial DNA concentrations for 16S rRNA sequencing.*

Adherence to New Jersey Department of Environmental Protection (NJDEP) sterile sampling methods was maintained, and the Volume-Averaged Purging and Sample Collection method (New Jersey Department of Environmental Protection [NJDEP], 2005) was utilized for this project. Wells were purged using a submersible pump, which was decontaminated before purging at each well. At each location, the pump was positioned one foot above the screened or open hole interval and, where well productivity allowed, three well volumes were purged. In wells with poor productivity, purging was halted when the water level dropped to the pump intake. The well was then allowed to recharge, a minimum of 1 h, prior to collecting analytical samples. Flow rate, volume purged, and water quality parameters including ORP, pH, temperature, turbidity, specific conductivity, and dissolved oxygen were recorded at the beginning of purging, after each well volume extracted, and at the time of sample collection.

A new Teflon^®^ lined bailer was lowered twice for analytical sampling at each location, first for VOC analyses, then again to collect additional volume for microbial analysis. To increase microbial density, the bailer was used to agitate sediments at the bottom of the well before being brought to the surface for collection.

### Water Chemistry

For each quarterly sampling event, water chemistry parameters were assessed both in the field and in the laboratory. Parameters to assess geochemistry include pH and dissolved oxygen. Water quality parameters were collected using a using an YSI 556 series multi-meter nested within a flow through cell, except at the time of analytical sample collection, when a flow through cell was not used. DCM and other volatile organic compounds were analyzed in the laboratory by Gas Chromatograph/Mass Spectrometry using EPA method 624 (EPA, 1984).

### Sample Preparation and DNA Extraction

Groundwater samples underwent centrifugation at 10,000 revolutions/minute for 10 min using a Sorvall RCB5 Superspeed Centrifuge (Thermo Scientific, Waltham, MA, United States). The supernatant from each sample was discarded, and the pellet was transferred with a scalpel to a sterile 10 ml Falcon tube (Corning Life Sciences, Tewksbury, MA, United States). Nucleic acid extractions were performed on approximately 0.25 g of each sediment pellet (*n* = 26) using a MoBio Powersoil DNA Isolation kit following the manufacturer’s instructions (MoBio Carlsbad, CA, United States). The cell-disruption step was performed using the Disruptor Genie cell disruptor (Scientific Industries, Bohemia, NY, United States). The resulting genomic DNA was eluted in 50 μL of 10 mM Tris.

### Acridine Orange Direct Counts

Samples were filtered through a 0.2 μm pore size black polycarbonate membrane (Whatman International Ltd., Piscataway, NJ, United States). Filtered cells were stained with 25-mg/ml acridine orange for 2 min in the dark. Unbound acridine orange was filtered through the membrane with 10 ml filter sterilized 1X PBS (Sigma–Aldrich Corp., St. Louis, MI, United States) and the rinsed membrane was mounted on a slide for microscopy. Cells were imaged with a FITC filter on a Zeiss Axioskop microscope (Carl Zeiss, Inc., Germany).

### 16S rRNA Library Preparation and Gene Sequencing

Amplification of the 16S rRNA gene was performed via Illumina Polymerase Chain Reactions (PCR), using an MJ Research PTC-200 thermocycler (Bio-Rad, Hercules, CA, United States) and carried out with the following cycling conditions: 98°C for 3 min, followed by 35 cycles of amplification (98°C for 1 min, 55°C for 40 s, and 72°C for 1 min), then 72°C for 10 min and kept at 4°C. Pooled PCR products were gel purified using the Qiagen Gel Purification Kit (Qiagen, Frederick, MD, United States) and then quantified using the Qubit 2.0 Fluorometer (Life Technologies, Carlsbad, CA, United States). Prior to submission for sequencing, libraries were validated using the 2100 Bioanalyzer DNA 1000 chip (Agilent Technologies, Santa Clara, CA, United States). Pooled libraries were stored at -20°C until they were shipped on dry ice to the California State University Northridge for sequencing. Library pools were size verified using the Fragment Analyzer CE (Advanced Analytical Technologies Inc., Ames, IA, United States) and quantified using the Qubit High Sensitivity dsDNA kit (Life Technologies, Carlsbad, CA, United States). Sequencing was performed using the Illumina MiSeq v2 Reagent Kit with 16S rRNA library sequencing primers and set for 150 base, paired-end reads.

### Bioinformatics and Statistical Analyses

Sequences were trimmed at a length of 150 bp and quality filtered at an expected error of less than 1% using USEARCH v7 (Edgar, 2013). After quality filtering, reads were analyzed using the QIIME 1.9.0 pipeline (Caporaso et al., 2010, 2011). Chimeric sequences were identified using USEARCH61 (Edgar, 2010). A total of 1.7 million sequences were obtained after quality filtering and chimera checking. Open reference operational taxonomic units (OTUs) were picked using the USEARCH61 algorithm (Edgar, 2010), and taxonomy assignment was performed using the Greengenes 16S rRNA gene database (13-5 release, 97%) (DeSantis et al., 2006). Assigned taxonomy were organized into a BIOM formatted OTU table, which was summarized within QIIME 1.9.0. OTUs that were not classified at the kingdom taxonomic rank were discarded. Additionally, 18 potential DCM-degrading OTUs identified in previous literature were summarized and quantified within each DCM concentration grouping. Relative abundances were organized in a table format. Relative abundances of the 10 most abundant potential DCM-degrading bacteria were plotted within Microsoft Excel.

Alpha diversity rarefaction curves were generated within the QIIME 1.9.0 sequence analysis package using an unrarified OTU table. Multiple rarefactions were conducted on sequences across all samples from minimum depth of 0 sequences, to a maximum depth of 8,000 sequences, with a step size of 1,000 sequences/sample for 20 iterations. Alpha rarefactions were then collated and plotted using Heip’s Evenness and observed species richness metrics. Alpha diversity was compared between DCM concentration groupings as well as sampling well. Alpha diversity comparisons were conducted using a two-sample *t*-test and non-parametric Monte Carlo permutations (*n* = 999).

Beta diversity was calculated using the weighted UniFrac distance metric and visualized with Principle Coordinates Analysis (PCoA) in EMPeror (Vázquez-Baeza et al., 2013). Analysis of Similarity (ANOSIM) was used to test differences in community structure within the samples (1000 Monte Carlo permutations) grouped by sampling location. Adonis tests were performed on weighted UniFrac distance matrices to determine the amount of variation explained by sampling well and measured continuous parameters. Alpha levels of 0.05 were used to detect significance of categorical or continuous variables. Directional PCoA plots were plotted with samples distributed along the *x*-axis based on increasing DCM concentrations that underwent log + 1 transformation.

Variability of sample distribution within Euclidean space was assessed through the calculation of average weighted UniFrac distance between samples within the same DCM concentration grouping. Spearman correlations were calculated in R studio to examine the relationship between continuous water chemical variables and taxa abundance. Kruskal–Wallis tests for significance were calculated using an unrarified OTU table within QIIME with “DCM grouping” chosen as the comparison category to reveal enriched taxa in each concentration group. Taxa unclassified at the kingdom level were not shown.

A row containing DCM concentrations for each sample was appended to the bottom of a CSS normalized OTU table. The heatmap.2 function within the gplots package for R Studio was used to generate an abundance heatmap. Dendrogram plotting was turned on to include the clustering of the samples and genera.

Operational taxonomic unit networks were generated within QIIME 1.9.0 from an OTU table rarified to a sequencing depth of 3000 sequences per sample to account for differential sequencing bias. OTUs unclassified at the genus taxonomic rank were removed. Networks were plotted and visualized using the AllegroLayout plugin within the data visualization tool Cytoscape-3.2.1. DCM degrading taxa of interest were highlighted manually. A Co-occurrence network was created with the Cytoscape plugin Conet. An unrarified OTU table was uploaded into the program with DCM-concentration selected as the metadata feature. Parameters where set to reveal co-occurrence patterns that correlated above a 0.9 Spearman’s test to reveal the 100 top and bottom most results. A command line was generated from the Conet Cytoscape plugin and executed in a command prompt. A GDL network created by the command line was uploaded into the Conet plugin to complete visualization. Nodes of interest where then selected and a separate network was created from the nodes of interest and their immediate neighbors.

## Results

### Groundwater Chemistry

Distinct differences in measured water chemical parameters were observed between groundwater samples collected in this study. DCM concentration (μg/L) spanned seven orders of magnitude (0.89–9,800,000 μg/L) and displayed the greatest difference amongst measured variables within the groundwater (**Table 1**). pH ranged from 4.44 to 8.40 units. Samples were grouped by increasing DCM concentration for alpha and beta diversity analysis in the following manner: 0–10 μg/L (*n* = 10), 10–100 μg/L (*n* = 5), 10,000–100,000 μg/L (*n* = 4), 100,000–1,000,000 μg/L (*n* = 3), and 1,000,000–10,000,000 μg/L (*n* = 4). Though cell counts revealed higher biomass concentration (1.50E + 07 to 1.44E + 08 cells/ml) in groundwater with DCM concentrations <100 μg/L compared to cell counts (3.76E + 06 to 1.86E + 07 cells/ml) from samples >10,000 μg/L, differences were not statistically significant (*p* > 0.05) (Supplementary Table S1).

### Microbial Community Diversity

Species richness analysis of the 16S rRNA gene sequences revealed a robust bacterial community existed in the groundwater at this site. Alpha diversity analysis of observed taxa resulted in 274–984 unique bacterial Operational Taxonomic Units (OTUs >97%) within the groundwater samples (Supplementary Figure S1). Groundwater with DCM concentrations <10 μg/L possessed nearly the same number of observed OTUs (observed species = 528; *n* = 10) compared to samples with >1,000,000 μg/L DCM (observed species = 487; *n* = 4), but differences were not significant (*p* > 0.05). No significant differences between any two DCM concentration groupings were observed when comparing both species richness and evenness. Alpha rarefaction curves appeared to reach a horizontal asymptote as sampling depth increased, indicating the level of sequencing conducted nearly saturated observed diversity.

**FIGURE 2:**
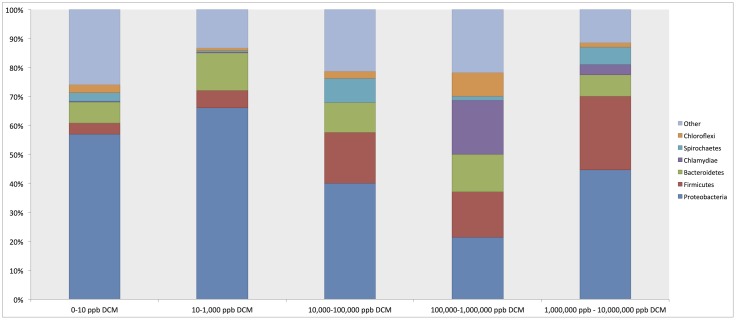
Relative abundance summaries of bacterial phyla reveal differences in general microbial community structure in groundwater samples grouped by increasing dichloromethane (DCM) concentration (*n* = 26). Plots were generated from an unrarified OTU table within QIIME 1.9.0. The 6 most abundant phyla across all samples are shown, with remaining taxa grouped in an “other” category. Shifts in general bacterial community structures in response to increased DCM concentration were observed. Proteobacteria were identified as the most abundant phylum across all samples, with average relative abundances comprising a range of 21.4–57% of the bacterial community within each group. A decrease in the average relative abundance of Proteobacteria was observed in samples with >10,000 μg/L DCM. Conversely, a rise in the Firmicutes abundance can be observed in samples with >10,000 μg/L DCM. A distinct spike in the Chlamydiae was noted in samples >100,000 μg/L DCM. Shifts in phylum relative abundances serve as a primary indication of microbial community responses to increased DCM concentration.

Phylum-level taxonomic assignments of 16S rRNA gene sequences revealed shifts in overall bacterial community structure with respect to DCM concentration (**Figure 2**). Proteobacteria were the most abundant phylum across all DCM concentration groupings, ranging from 21.4 to 67% of sample composition. Proteobacteria sequence abundance decreased 25.9% in samples with higher (>1000 μg/L) DCM concentrations when compared to samples with lower (<1000 μg/L) DCM concentrations. Conversely, Firmicutes were 13.9% more abundant in samples with DCM concentrations >1,000 μg/L when compared to samples with <1,000 μg/L DCM. The Bacteroidetes did not vary with respect DCM concentration. Additionally, average relative abundances for Chlamydiae in sample groups with DCM concentrations ranging from 100,000–1,000,000 μg/L and 1,000,000–10,000,000 μg/L were 18.6 and 3.4%, respectively, but only account for 0.3% of the total microbial community structure of samples with DCM concentrations <1000 μg/L.

**FIGURE 3:**
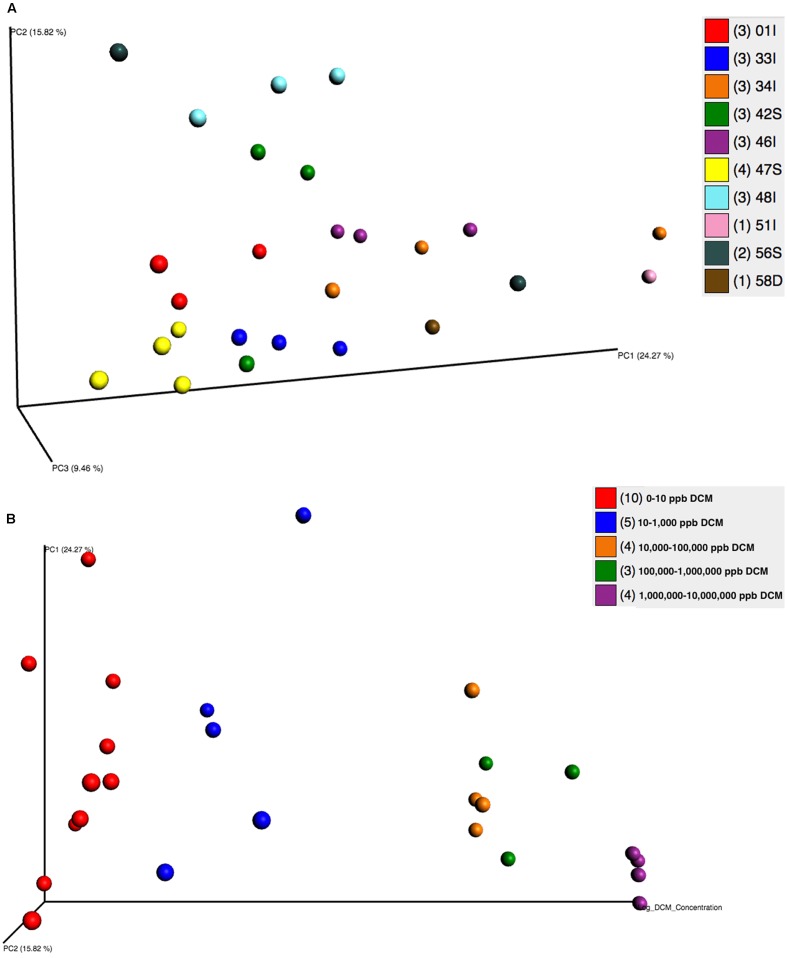
**(A,B)** Principal Coordinates Analyses (PCoA) plots were utilized to visualize differences in microbial community structure between groundwater samples grouped by well **(A)** and DCM concentration **(B)**. PCoA plots were generated from a weighted UniFrac distance matrix calculated within QIIME 1.9.0. **(A)** Samples are colored based on sampling well. Distinct clustering of samples can be observed within wells 01I (red), 33I (blue), 46I (purple), 47S (yellow), and 48I (teal). Sample clustering based on well was considered to be significant (ANOSIM, *p* = 0.001) and accounted for 55% of variation in microbial community structure between samples (adonis *p* = 0.001). **(B)** A directional PCoA plot distributes samples along the *x*-axis by increasing DCM concentrations that underwent log + 1 transformations. Clustering of samples with higher DCM concentrations can be observed. DCM concentration (μg/L) was found to cause significant shifts (10%, adonis *p* = 0.004) in microbial community structure. Variability between samples significantly decreases as DCM concentrations rise (Z score = 3.17, Mann–Whitney U P1 = 0.0009, P2 = 0.015), indicative of a shaping toward a defined microbial community structure as DCM increases.

Beta diversity analyses revealed significant clustering by sampling location and DCM concentration, as visualized by principal coordinates analysis (PCoA) plots (**Figures 3A,B**). PCoA plots showed discrete bacterial community structures within wells 47S, 01I, 33I, 46I, and 48I (ANOSIM, *p* = 0.001) (**Figure 3A**). Sampling location explained the most (55%) variation in bacterial community structure between samples (Adonis, *p* = 0.001). Directional PCoA plots showed a strong relationship between DCM concentration and bacterial community composition, as exemplified by clustering of samples with DCM concentrations >10,000 μg/L (**Figure 3B**). DCM concentration explained 10% of variation in beta diversity between samples (Adonis, *p* = 0.004). Samples with DCM concentrations ranging from 1,000,000–10,000,000 μg/L formed a distinct cluster from the remaining cohorts at the bottom of the PC1 axis, and to the back of the PC2 axis. Greater phylogenetic variation was observed between samples with DCM concentrations <1,000 μg/L in comparison to samples with >10,000 μg/L. Samples with >10,000 μg/L DCM observed significantly less variation between samples when compared to the <1,000 μg/L DCM cohort (*Z* score = 3.17, Mann–Whitney U P1 = 0.0009, P2 = 0.015).

Of all collected continuous water chemistry data, DCM concentration was found to correlate strongly (spearman’s rho >0.65) with the highest number of microbial taxa. A total of 16 taxa, including known degraders such as *Dehalobacterium*, *Acetobacterium*, and *Desulfovibrio aminophilus* had strong positive correlations with DCM concentration (**Table 2**). Many taxa possessing strong positive correlations with DCM concentration were unclassified beyond the family taxonomic rankings, including Unclassified Ruminococcaceae, Unclassified Elusimicrobiales, TM7-[Blgi18], Unclassified Bacteroidales and Unclassified Endomicrobia. Two OTUs assigned to the *Methylomonas* genus were the only taxa negatively correlated with DCM concentration (**Table 2**). Dissolved oxygen (DO) (mg/L) was found to correlate positively with a total of 4 unique OTUs (*p* > 0.65), including Pirellulaceae, Ruminococcaceae, Chloroflexi Ellin 6529, and Gemmatimonadetes KD8-87 (Supplementary Table S2). A summary of all strong correlations with measured water chemical parameters can be found in Supplementary Table S2. pH fluctuated between sampling wells, with site 01I possessing the lowest average pH (5.25) and well 58D possessing the highest average pH (8.11). Well 46I possessed the highest average pH (7.69) of all sampling wells with at least 3 samples included in our 16S investigation. Within well 01I, the *Geobacter, Magnetospirillum*, and the *Dehalobacter* were the 3 most abundant genera, with relative abundances at 13.26, 13.12, and 13.04% respectively. Within well 46I, the *Hydrogenophaga*, *Cloacibacterium*, and *Hymenobacter* were most abundant, with relative abundances at 15.08, 9.17, and 7.54%, respectively.

**Table 2:** Spearman’s non-parametric correlation results of bacterial abundance data and DCM concentration.

Spearman’s rho value	*p*-value	Taxonomy
-0.76	7.50E-06	Methylomonas
0.75	1.25E-05	Unclassified Ruminococcaceae
0.74	1.55E-05	[Blgi18]
0.73	1.94E-05	Dehalobacterium
0.72	3.66E-05	Unclassified Elusimicrobiales
0.70	7.25E-05	Unclassified Acetobacterium
0.69	0.000110041	Unclassified Bacteroidales
0.68	0.000121371	Desulfovibrio aminophilus
0.67	0.000166509	[Blgi18]
0.67	0.00020055	Unclassified Endomicrobia
0.66	0.000225836	Treponema
-0.65	0.000334437	Methylomonas
0.65	0.000348058	Treponema
0.65	0.000360755	Unclassified Ruminococcaceae
0.65	0.000362283	Desulfurispora
0.65	0.000362283	Alkalibacterium


**FIGURE 4:**
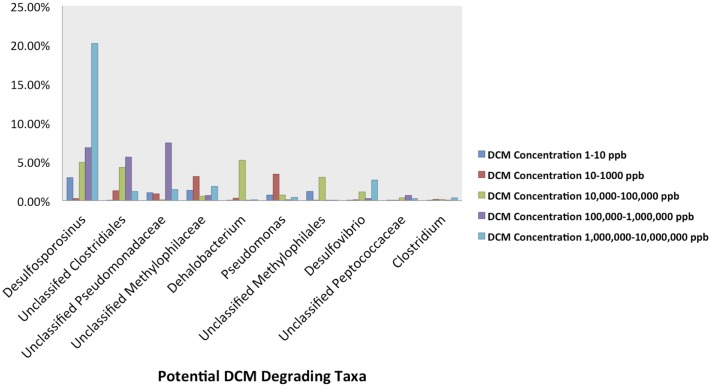
Abundance plots present the relative abundances of the 10 most abundant potential DCM-degrading taxa of interest within the groundwater samples grouped by DCM concentration. Plots were generated from an unrarified OTU table, and present the average relative abundances of 10 potential DCM degrading OTUs within each DCM concentration grouping. A unique DCM-degrading consortium was identified within each group of groundwater samples. Over 20% of the total 16S rRNA gene sequences within the most contaminated samples (1,000,000–10,000,000 μg/L DCM) were assigned to the *Desulfosporosinus* genus, more than double the relative abundance identified in any other group. This highly contaminated group also observed the largest relative abundance (2.6%) of *Desulfovibrio*, while samples with DCM concentrations from 100,000 to 1,000,000 μg/L DCM possessed a high average abundance (7.4%) of Unclassified Pseudomonadaceae. *Dehalobacterium* were observed in greatest abundance (5.1%) within samples with DCM concentrations ranging from 10,000 to 100,000 μg/L DCM. *Pseudomonas* sequences were most abundant in samples with lower DCM concentrations (100–1,000 μg/L DCM), with an average relative abundance of 3.4%. Samples with the lowest DCM concentrations (0–10 μg/L DCM) were the only set of groundwater samples that was not dominated by any specific known DCM-degrading taxa. The data suggests that different levels of DCM may promote a unique environment conducive to niche-specific DCM-degrading taxa.

The relative abundance of potential DCM-degrading bacteria increased with groundwater DCM concentration. The total relative abundance of these potential DCM-degrading taxa increased from 7.5 to 28.6% from the least (0–10 μg/L DCM) to most (1,000,000–10,000,000 μg/L DCM) contaminated samples. Different DCM-degrading assemblages emerged within the differentially contaminated samples (**Figure 4**). In samples with >1,000,000 μg/L DCM, over 20% of the total 16S rRNA gene sequences were assigned to the *Desulfosporosinus* genus, more than double the relative abundance identified in any other DCM group. These highly contaminated samples also had the largest relative abundance (2.6%) of *Desulfovibrio*, while samples with DCM concentrations ranging from 100,000–1,000,000 μg/L DCM possessed the highest abundance (7.4%) of Unclassified Pseudomonadaceae 16S rRNA gene sequences in comparison to all other DCM concentration groupings. *Dehalobacterium* were observed in low abundance (<1%) across all DCM concentration groupings, except for samples with 10,000–100,000 μg/L DCM, in which it composed 5.1% of the microbial community. *Pseudomonas* sequences were most abundant in samples with DCM contamination ranging from 10 to 1,000 μg/L, with an average relative abundance of 3.4%. Samples with less than 10 μg/L DCM were not dominated by any known DCM-degrading taxa.

**FIGURE 5:**
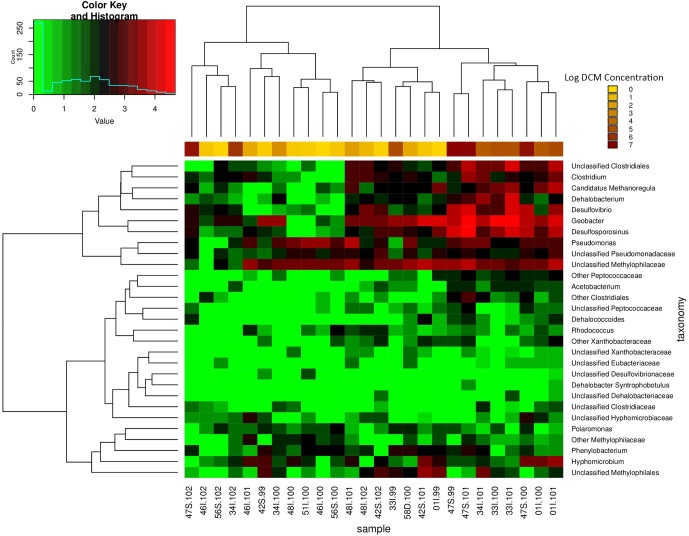
Relative abundance heatmap of potential DCM-degrading biomarker taxa. This heatmap displays the abundance of 29 taxa previously associated with growth on DCM or similar contaminants within each groundwater sample (*n* = 26). Taxa of high abundances are highlighted in red, whereas taxa of low abundance are highlighted in green. Samples with higher DCM concentrations possess the highest abundances of biomarker taxa of interest, with as much as a four order of magnitude difference in abundance in comparison to samples of lower DCM concentration. As a result, samples of high DCM concentration appear to cluster together on the right side of the plot. Unclassified Clostridiales, *Clostridium*, *Candidatus Methanoregula*, *Dehalobacterium*, *Desulfovibrio*, *Desulfosporosinus*, *Pseudomonas*, and Unclassified Methylophilaceae are present in the highest abundance within the high DCM concentration samples. The data suggest that the presence and abundance of the potential DCM degrading taxa of interest may be driving the observed shift in microbial community structure with increased DCM concentration. A majority of the anaerobic DCM degraders are observed in higher within the samples with higher levels of contamination, but do not thrive within samples with lower contamination. Clustering of samples further supports the finding of niche-specific DCM degrading assemblages within samples of similar DCM concentrations.

Sampling wells 34I and 01I observed the greatest variation in DCM concentration in comparison to all remaining wells (**Table 1**). A heatmap of potential DCM-degrading assemblages within these sites revealed fluctuations in DCM degrading assemblages within both sampling locations (Supplementary Figure S2). Clustering of samples when considering DCM degrading OTUs within 34I and 01I appears to be driven by DCM concentration, rather than sampling location, as samples of lower DCM concentration are clustered to the right of the heatmap with lower counts of DCM degrading assemblages. The relative abundance of *Desulfosporosinus* increases within well 01I from 1.5 to 6.99% in response to increased DCM concentration. Within well 34I, an increased abundance (3.1%) of *Dehalobacterium* can be observed within sample 34I-101 (46,000 μg/L DCM). Samples 34I-100 (690 μg/L DCM) and 34I-102 (790,000 μg/L DCM) yielded relative abundances of *Dehalobacterium* below 0.5%. Both of these trends mirror fluctuations in DCM degrading taxa abundances when considering the entire dataset (**Figure 5**).

A total of 42 OTUs were enriched in samples with DCM concentrations above 10,000 μg/L DCM (Kruskal–Wallis *p* < 0.004) (Supplementary Table S3). When considering DCM concentration groupings 0–10 μg/L DCM and 10–100 μg/L DCM, no significantly enriched bacterial taxa were identified (Supplementary Table S3). A two-way heatmap presenting the relative abundance of potential DCM-degrading biomarker taxa displayed clustering of samples with DCM concentrations >10,000 μg/L (**Figure 5**). The relative abundance 16S rRNA gene sequences matching to potential DCM-degrading biomarker taxa were highest in samples of DCM concentration >10,000 μg/L, with as much as a four-fold increase in their relative percentage of the total community in comparison to samples of DCM concentration <1,000 μg/L, including *Dehalobacterium* and *Desulfosporosinus*. The enrichment in putative DCM-degrading taxa in more contaminated samples is further supported by the core microbiome analysis, which reveals the presence of 40 core OTUs that have been identified as DCM-degraders in highly contaminated samples (DCM concentrations >10,000 μg/L DCM), as compared to only five potential DCM-degrading core OTUs within less contaminated samples (DCM concentration <1,000 μg/L DCM (Supplementary Figure S3).

**FIGURE 6:**
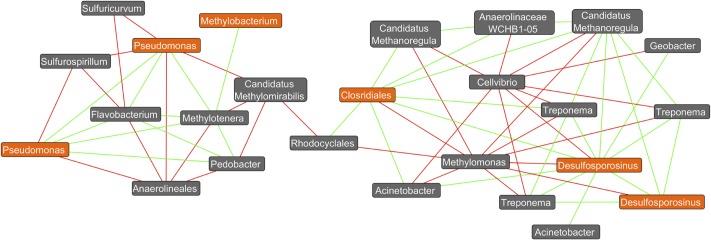
Co-occurrence network plot reveals strong positive and negative correlations (Spearman’s rho >0.9) between OTU abundances. A co-occurrence network generated from an unrarified OTU table containing bacterial abundance data from all 26 groundwater samples was created within the Cytoscape plugin Conet. All taxa unassigned at the kingdom taxonomic ranking are not shown. Only strong correlations (spearman’s rho >±0.9) were included in the network. Potential DCM-degrading taxonomy are highlighted in orange, with all remaining taxa highlighted in gray. Green edges connecting two nodes are indicative of a strong correlation (spearman’s rho >0.9) between the taxa, whereas red edges are indicative of a strong negative correlation (spearman’s rho < –0.9). It can be observed that 2 distinct clusters form at the right and left of the plot, with aerobic DCM degrading bacteria (Pseudomonas and *Methylobacterium*) identified in the left cluster, and potential anaerobic degraders (*Desulfosporosinus* and Clostridiales) on the right. Strong positive correlations can be observed between the potential DCM-degrader *Desulfosporosinus* and three unique *Treponema* OTUs in addition to *Candidatus Methanoregula*, *Acinetobacter*, *Geobacter*, and unclassified Clostridiales. *Methylomonas* and *Cellvibrio* were the only taxa calculated to have strong negative correlations (spearman’s rho <–0.9) with *Desulfosporosinus*. The unclassified Clostridiales taxon was also found to correlate strongly with *Treponema*, in addition to six other non DCM-degrading OTUs. Two unique OTUs assigned to the pseudomonas genus were found to have strong positive correlations with *Flavobacterium*, *Pedobacter*, and *Methylotenera*, and strong negative correlations with *Sulfurospirillium*, unclassified Anaerolineales, *Candidatus Methylomirabilis* and *Sulfuricurvum*. Potential DCM-degraders appear to correlate with specific bacteria not yet associated with DCM degradation, revealing possible degradation capacities or bacterial enrichment capabilities.

Co-occurrence network analysis revealed a variety of positive and negative correlations amongst members of these complex bacterial communities. For example, several positive interactions exist between putative DCM-degrading assemblages and bacteria yet to be associated with DCM degradation (**Figure 6**). Interestingly, two distinct sub-networks were observed, which map to both aerobic DCM degrading taxa (*Pseudomonas* and *Methylobacterium*) anaerobic degraders (*Desulfosporosinus* and Clostridiales). Two unique *Pseudomonas* OTUs as well as a *Methylobacterium* OTU had strong positive correlations with both *Flavobacterium* and *Methylotenera*, while both *Pseudomonas* OTUs also had strong negative correlations with *Sulfurospirilium* and *Anerolineales*. OTUs assigned to the *Desulfosporosinus* genus formed strong positive correlations (Spearman’s rho >0.9) with *Treponema, Candidatus Methanoregula*, *Acinetobacter*, *Geobacter*, and unclassified Clostridiales. *Methylomonas* and *Cellvibrio* were the only taxa calculated to have strong negative correlations (Spearman’s rho <-0.9) with *Desulfosporosinus*. The unclassified Clostridiales were also found to correlate strongly with *Treponema*, *Candidatus Methanoregula*, Rhodocyclales, and *Acinetobacter* OTUs.

## Discussion

Bacterial community composition was investigated in groundwater collected from a site undergoing natural attenuation to treat a DCM source area and dissolved phase plume in a fractured bedrock. High throughput sequencing of the 16S rRNA gene allowed for comprehensive analysis of bacterial community structure associated with DCM-contaminated groundwater. While there was little observed variability in bacterial concentration and richness across the groundwater samples, distinctive shifts in bacterial community composition were observed with respect to the DCM concentration. Bacterial community analyses of these groundwater environments revealed a surprisingly robust and diverse community within these DCM-contaminated environments. The observed bacterial community structure was shaped by variation in DCM concentrations, and putative novel taxa that correlate with DCM concentration were identified in these groundwater environments. This study represents the first community-wide assessment of *in situ* bacterial community diversity in DCM-contaminated groundwater using high-throughput sequencing technologies.

Alpha diversity analyses revealed a robust bacterial community within even the most highly DCM-contaminated groundwater samples, and consequently species richness did not change significantly between DCM concentration groupings. Previous research has found similarities in alpha diversity between groundwater samples with varying levels of trichloroethylene (TCE) (Lee et al., 2012) and total chlorine (Imfeld et al., 2008; Wang et al., 2014; Stanish et al., 2016) indicating that the indigenous microbial community is capable of withstanding and even utilizing chlorinated hydrocarbons in highly contaminated scenarios. Other molecular studies that have measured community-wide responses to DCM, have also suggested the existence diverse microbial communities associated with DCM-impacted environments (Leisinger et al., 1994; Dojka et al., 1998; Vuilleumier et al., 2001; Muller et al., 2011; Justicia-Leon et al., 2012). While hydrocarbon release into groundwater has been cited to cause a brief reduction in species richness directly after a spill, longitudinal studies show no significant reduction in diversity over time (Röling et al., 2002; Hernandez-Raquet et al., 2006; Alonso-Gutiérrez et al., 2009; Abed et al., 2014).

The lower biomass in higher concentration DCM groundwater samples could in part be due to toxicity of DCM and its conversion. The bacterial toxicity of DCM transformation has been previously described (Thier et al., 1993; Gisi et al., 1999; Kayser and Vuilleumier, 2001).

Previous work has also documented the toxicity to methanotrophic populations at DCM concentrations above 780 μM (Byers and Sly, 1993). While cell concentrations were slightly lower in highly contaminated groundwater samples, alpha diversity estimates revealed a robust bacterial community still persisted, even at DCM concentrations above 9.8 million μg/L. Future work, including metatranscriptomics, will enable the investigation of the activity of these bacterial assemblages within contaminated groundwater, revealing which organisms can survive and possibly contribute to the active degradation of DCM.

Beta diversity analyses assessed differences in phylogenetic distance between the samples and revealed a distinct bacterial community associated with samples with elevated DCM groundwater concentrations (**Figures 3A,B**). Chlorinated organics have been found to shape microbial communities in sediment (Lee et al., 2012; Hamonts et al., 2014; Atashgahi et al., 2016) and groundwater (Dojka et al., 1998; Park and Noguera, 2004; Zaa et al., 2010; Lee et al., 2012; Rossi et al., 2012; Kao et al., 2016). Variation in bacterial community structures between sampling wells was also, in part, related to differences in geochemical parameters between the sampling wells as variance-partitioning analysis indicated. Our findings are congruent with previous literature describing the role of ecological-niche factors such as pH, salinity, and temperature in determining microbial community composition and function (Park and Noguera, 2004; Xu et al., 2010; Wang et al., 2012; Bougouffa et al., 2013; Saleem, 2015; Techtmann et al., 2015). Variability in geochemistry had a significant impact not only on microbial community structure, but the potential mechanism of DCM-degradation as well. Near the source contamination area, in samples with the highest levels of DCM contamination, the mechanisms are predominantly anaerobic, indicative of an environment with less available free-oxygen as a result of DCM saturation. Elevated abundances of anaerobic bacteria including *Desulfosporosinus, Desulfovibrio*, and unclassified Clostridiales taxa can be observed in these highly contaminated sites (**Figure 4**). Conversely, increased abundance of aerobic degraders, such as the *Pseudomonas*, was noted in lower DCM concentration environments (**Figure 5**). This suggests that DCM degrading organisms are abundant throughout the source area and dissolved phase plume, and that different bacterial assemblages appear to be establishing niches within specific DCM concentration ranges.

Several putative DCM-degrading taxa were significantly enriched in specific groundwater environments. Well-cited DCM degrading taxa including *Dehalobacterium* and unclassified Methylophilaceae, were highly abundant across groundwater samples ranging from low to high levels of DCM concentration (**Figures 4**, **5**), have both been previously been found to degrade DCM via a fermentative pathway (Mägli et al., 1998; Justicia-Leon et al., 2012). Additionally, *Desulfovibrio* and *Acetobacterium* correlated strongly with higher DCM concentrations, and have been shown to grow rapidly in co-culture during DCM-exposure (Magli et al., 1995). Both the *Desulfovibrio and Acetobacterium* are genera of anaerobic bacteria, further indicating a shift toward anaerobic degradation in response to increased DCM concentration (Balch et al., 1977; Madigan, 2012). The sulfate-reducing *Desulfosporosinus* genus was most abundant (20.14%) genus within the most contaminated groundwater samples environments (>1,000,000 μg/L). This genus is known to possess genes required for chloroalkane degradation and have been isolated from groundwater sites contaminated with gasoline, but has not been cited as a specific DCM degrading taxa (Stackebrandt et al., 2003). Both the *Acetobacterium* and *Desulfosporosinus* fall within the Firmicutes, thus explaining the increased abundance of this phylum within highly contaminated DCM environments. Previous literature has shown that bacteria within the Firmicutes can utilize DCM as a sole carbon source under anoxic conditions (Kleindienst et al., 2017). The second most abundant genus within the highly contaminated samples, the *Sulfurospirillum*, (10.7%), has yet to be cited as a DCM-degrading taxon, but is a nitrate-degrader known to compete with sulfate-reducing bacteria, such as the *Desulfosporosinus*, for degradable oil organics (Hubert and Voordouw, 2007). Two other well-cited DCM-degraders, *Pseudomonas* and *Hyphomicrobium*, were observed in high abundance across all samples, including DCM rich-samples (>1,000 μg/L), and are capable of aerobic (Guo, 1990; Vuilleumier et al., 2001; Krausova et al., 2006) and facultatively anaerobic (Vuilleumier et al., 2001; Nikolausz et al., 2005) degradation, respectively. The *Geobacter* were abundant (13.26%) within well 01I, a site with the lowest average pH reading (5.25) across all sampling wells. *Geobacter* are known neutrophiles, and have been found to thrive in low pH conditions, where they can mediate ferric iron reduction (Johnson et al., 2012). The *Hydrogenophaga* were identified in greatest abundance within well 46I, found to possess the highest average pH (8.11). This genus has been found to grow optimally at pH <7.0, and are capable of mineralizing methyl tertbutyl ether in groundwater (Streger et al., 2002; Yoon et al., 2008).

Other bacterial taxa also positively correlated with DCM concentration but are not yet associated with DCM degradation, suggesting possible degradation capacities or supportive roles. Interestingly, two observed clusters of taxa appear to separate potential aerobic and anaerobic DCM-degrading taxa, indicative of two differential networks of bacteria capable of degradation in oxygenated or anoxic groundwater (**Figure 6**). The aerobic genus *Pseudomonas* has been found to utilize DCM as a secondary substrate (LaPat-Polasko et al., 1984; Krausova et al., 2006) and positively correlate with *Methylotenera* and *Flavobacterium* (Bernardet et al., 1996; Kalyuzhnaya et al., 2006). While *Flavobacterium* have yet to be associated with DCM degradation, they have been found to degrade alternative xenobiotics when grown in co-culture with *Pseudomonas* (Trzesicka-Mlynarz and Ward, 1995). *Methylotenera* has yet to be associated with DCM degradation as well (Kalyuzhnaya et al., 2006). However, species within the *Methylotenera* genus have been found to possess multiple genes required for formaldehyde activation and metabolism, a byproduct of aerobic DCM degradation in DCM rich environments (Marx et al., 2003; Bosch et al., 2009). Both *Methylotenera* and *Flavobacterium* are methylotrophic bacteria found to utilize methane, a byproduct of DCM degradation, *in situ* (Hesselsoe et al., 2005; Kalyuzhnaya et al., 2006; Justicia-Leon et al., 2012; Beck et al., 2013).

Bacteria including *Acinetobacter*, *Geobacter*, and *Treponema* exhibited significant positive correlations to the *Desulfosporosinus* (**Figure 6**). Both *Geobacter*, and *Treponema* members, which correlated positively to the Clostridiales taxa, have been previously found in groundwater contaminated with chlorinated organic compounds (Imfeld et al., 2010; Lovley, 2011; Miura et al., 2015). Specific OTUs not yet linked to DCM degradation may be enriched as a result of byproduct accumulation from such processes. Within anaerobic conditions, DCM is transformed to methane, carbon dioxide, and acetate during which sulfate, nitrate or ferric iron may act as the predominant electron acceptors for anaerobic DCM degrading taxa such as *Dehalobacterium* and *Desulfovibrio* (Magli et al., 1995; De Best et al., 2000; Vuilleumier et al., 2001). Facultative anaerobes such as *Hyphomicrobium* and *Actinetobacter* have been found to degrade DCM in both aerobic and anaerobic conditions, capable of growth with either nitrate or oxygen serving as the terminal electron acceptor (Vuilleumier et al., 2001).

Our bacterial community profiling of these groundwater environments using high throughput sequencing of the 16S rRNA gene revealed a distinct shaping of the autochthonous bacterial communities in response to varying DCM concentrations. Potential degraders of chlorinated organics were observed in greater abundances in samples with DCM concentrations >1000 μg/L. Future work such as bacterial isolation, would enable us to confirm if these potential novel DCM degrading taxa are able to actively degrade DCM. Additionally, while extrapolation of 16S rRNA gene data revealed that the groundwater samples have the potential to carry out chloroalkane and alkene degradation, future functional metagenomic and metatranscriptomic analyses will greatly benefit our understanding of the functional capacity of these bacterial communities. With such data, functional potential and expression patterns can help estimate the biodegradation potential of the microbial community. This would allow us to potentially identify novel DCM degrading taxa, as well as distinguish monitored natural attenuation as a useful bioremediation technique of DCM, which would serve as a cost efficient benefit to current remediation practices.

## Author Contributions

JW: was involved in both wetlab processing and bioinformatic analysis of all collected samples. Contributed to the writing of all sections of the manuscript. Gave several poster presentations as well as talks regarding the original research. VK: assisted with bioinformatics. Contributed writing for the methods section of the manuscript. WB: assisted with bioinformatics. Contributed writing for the methods and results section. Generated figures relating to network analysis. NU: Assisted with manuscript preparation and formatting and wetlab processing. Was integral in organizing references and assisting with manuscript revisions. CM: Assisted with wetlab processing and assisted with manuscript formatting. MC and TH: Assisted with sequencing and cell count analysis. Contributed to methods writing as well as results. TM: Assisted with writing every section of manuscript. Provided metadata information from the investigated site. DM: Assisted with writing every section of manuscript. Provided metadata information from the investigated site and managed chemical investigation of the site. JM: Was involved in sample collection and manuscript generation. RM: Assisted with sequencing/manuscript review. KR: Provided mathematical guidance for statistial analysis, assisted with methods. RL: Mentored all aspects of the original investigation, contributed writing to every section of the manuscript.

## Conflict of Interest Statement

The authors declare that the research was conducted in the absence of any commercial or financial relationships that could be construed as a potential conflict of interest.
