# Supplementary material for: Bacterial Community Dynamics in Dichloromethane-Contaminated Groundwater Undergoing Natural Attenuation
Source: Front Microbiol. 2017 Nov 22;8:2300. doi: 10.3389/fmicb.2017.02300 (PMC5702783; doi:10.3389/fmicb.2017.02300)
Supplement: Supplementary file 1 [file Data_Sheet_1.pdf]

Supplementary Material

Bacterial Community Dynamics in Dichloromethane-contaminated Groundwater Undergoing Natural Attenuation

Justin Wright[1,2], Veronica Kirchner[1], William Bernard[1], Nikea Ulrich[1], Christopher McLimans[1], Maria Fernanda Campa[3,7,8], Terry Hazen[3,4,5,6,7,8] PhD, Tamzen Macbeth[9] PhD, David Marabello[9], Jacob McDermott[9], Rachel Mackelprang[10] PhD, Kimberly Roth PhD[1], Regina Lamendella PhD[1,2 *]

[1]Lamendella Laboratory, Juniata College, Department of Biology, Huntingdon, PA, USA
[2]Wright Labs, LLC, Huntingdon, PA, USA
[3]Bredesen Center for Interdisciplinary Research and Graduate Education, University of Tennessee, Knoxville, TN.
[4]Department of Microbiology, University of Tennessee, Knoxville, TN
[5]Department of Civil and Environmental Engineering, University of Tennessee, Knoxville, TN
[6]Earth & Planetary Sciences, University of Tennessee, Knoxville, TN
[7]Biosciences Division, Oak Ridge National Laboratory, Oak Ridge, TN.
[8]Institute for a Secure and Sustainable Environment, Knoxville, TN.
[9]CDM-Smith, Edison, NJ, USA

[10]California State University Northridge, Department of Biology, Northridge, PA, USA *

Correspondence: Regina Lamendella: lamendella@juniata.edu

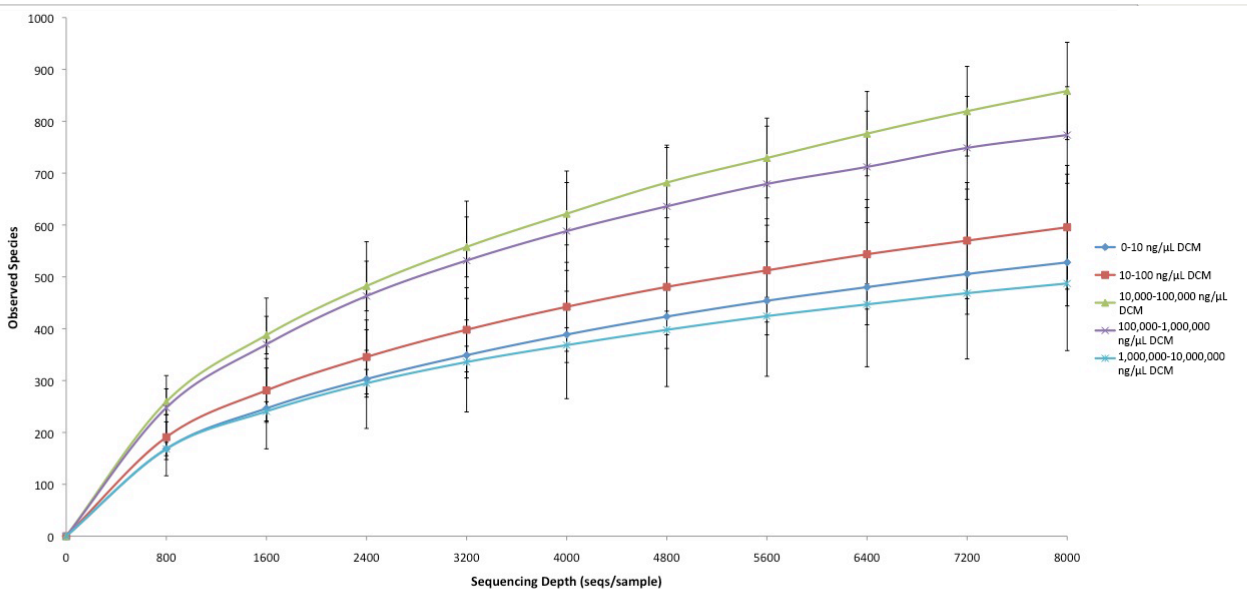

Figure S-1. Alpha Diversity rarefaction curves comparing observed species richness between DCM concentration groupings. Species richness was estimated by performing multiple rarefactions up to a depth of 8,000 sequences per sample, with a step size of 800 sequences and 20 iterations at each step. The richness of an unrarified OTU table was estimated using observed richness matrices, and were visualized using qiime-1.9.0. Each sample reaches an asymptotic curve as sequencing depth increases, suggesting a sufficient maximum sampling depth was utilized. No significant differences in species richness can be observed between any two DCM sample groupings.

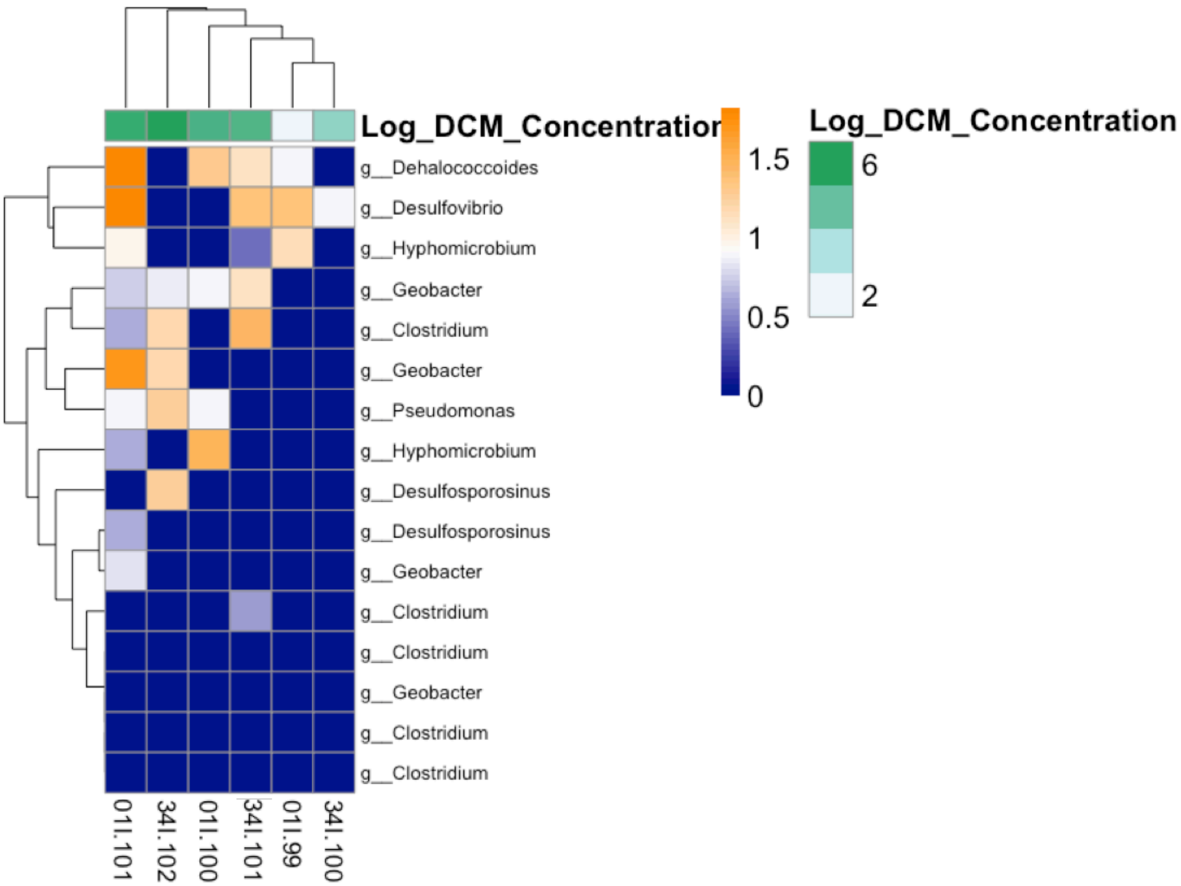

Figure S-2. Relative abundance heatmap of potential DCM degrading OTUs classified at the genus level within wells 34I and 01I. A relative abundance heatmap of potential DCM degrading OTUs was generated from a *Phyloseq* object and visualized within R Studio using the *Pheatmap* package. Samples within 34I and 01I were selected for comparative analysis, as DCM concentrations within these respective wells varied 4 orders of magnitude over time. Relative abundances of potential DCM degrading OTUs summarized at the genus level were selected for and included within the heatmap. Interestingly, clustering of samples appears to be driven by DCM concentration, rather than sampling well. Samples with the lowest DCM concentrations are observed to the right of the plot, and samples of increased DCM concentration can are clustered to the left. This serves as an indication of DCM concentration driving shifts in the relative abundance and prevalence of potential DCM degrading assemblages within the groundwater, even when considering extreme fluctuations within a defined sampling well.

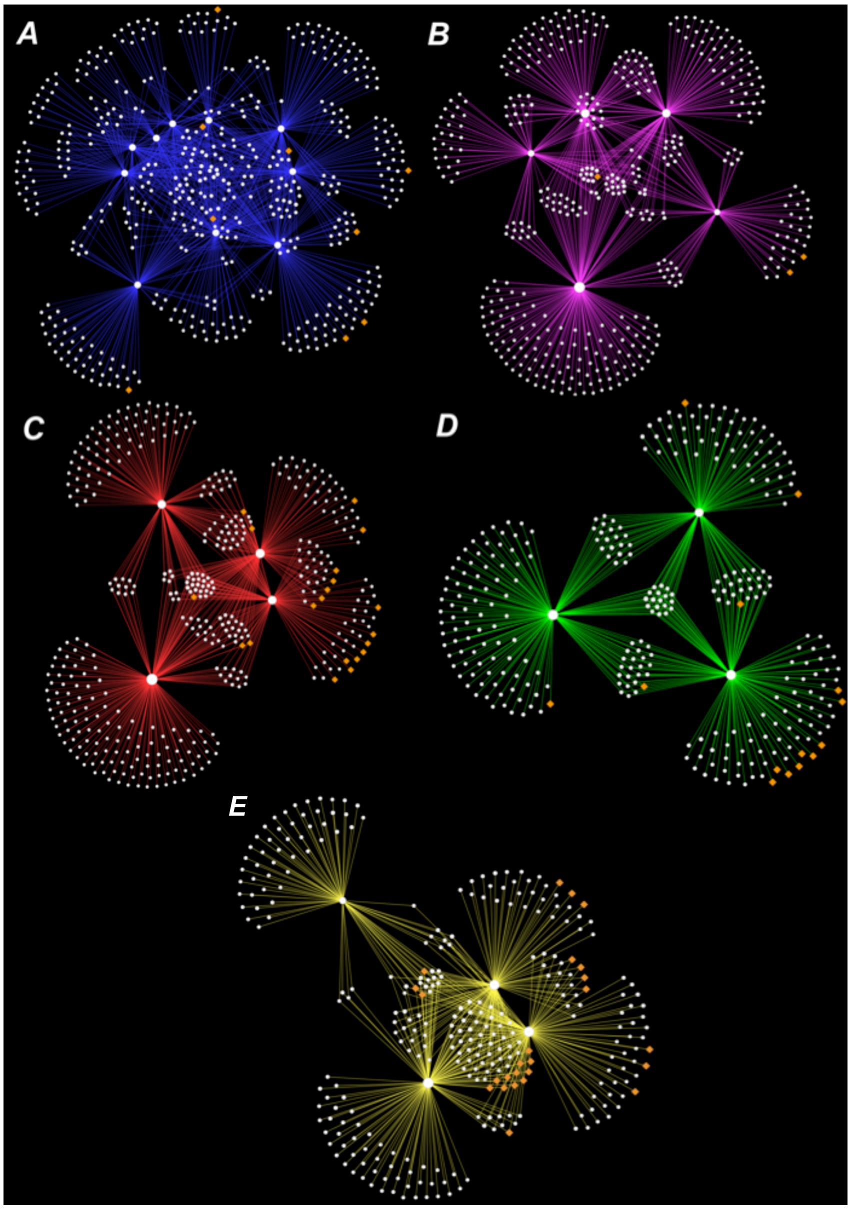

Figure S-3A-E. Network analysis plot reveals shared and unique OTUs between samples belonging to each respective DCM concentration grouping. Networks were plotted and visualized using the AllegroLayout plugin within the data visualization tool *Cytoscape-3.2.1.* OTUs identified at the genus taxonomic rank within each DCM concentration group are shown. The larger circular nodes are representative of groundwater samples within each DCM concentration cohort, the smaller circular nodes represent non-DCM degrading OTUs, and the orange square nodes represent potential DCM degrading OTUs. Each colored network includes samples from the same DCM concentration range: blue=0-10 μg/L DCM, purple=10-100 μg/L, red=10,000-100,000 μg/L DCM, green= 100,000-1,000,000 μg/L DCM, yellow= 1,000,000-10,000,000 μg/L DCM. A connection between an OTU and a sample is indicative of the presence of the OTU within its respective sample's microbial community. It can be observed that a total of 25 potential DCM degrading taxa were identified within the DCM concentration 1,000,000-10,000,000 μg/L (E) network. While some DCM degrading taxa are unique to individual samples, 19 are shared by at least two samples, and 14 are shared by three samples. A

total of 14 OTUs were shared between all 4 samples within Group E, 3 of which were potential DCM degrading taxa. Group E possesses the largest amount of DCM degrading taxa interconnected by 2 or more samples when compared to any other concentration grouping.

Supplementary Table 1. Acridine orange cell count data obtained from 5 groundwater samples.

Sample ID	Cell Counts	DCM Concentration
GW-47S-103	1.86E+07	High
GW-01I-103	3.76E+06	High
GW-42S-103	1.44E+08	Low
GW-34I-103	1.29E+07	High
GW-56S-103	1.50E+07	Low

Supplementary Table 2. Spearman's correlation calculations displaying strong (rho > |.65|) with continuous measured water parameters.

Depth (ft) Test Stat	pval	Phylum	Class	Order	Family	Genus
**-0.6409**	0.0003	*Proteobacteria*	*Betaproteobacteria*	*Rhodocyclales*	*Rhodocyclaceae*	*Dok59*
**-0.6205**	0.0005	*OD1*				
**DO (mg/L) Test Stat**	**pval**	**Phylum**	**Class**	**Order**	**Family**	**Genus**
**0.7195**	0.0000	*Planctomycetes*	*Planctomycetia*	*Pirellulales*	*Pirellulaceae*	*A17*
**0.6936**	0.0000	*Chloroflexi*	*Ellin6529*			
**0.6647**	0.0001	*Firmicutes*	*Clostridia*	*Clostridiales*	*Ruminococcaceae*	
**0.6525**	0.0002	*Gemmatimonadetes*	*Gemmatimonadetes*	*KD8-87*		
**Flow (Gal/min) Test Stat**	**pval**	**Phylum**	**Class**	**Order**	**Family**	**Genus**
**-0.6667**	0.0001	*Bacteroidetes*	*Bacteroidia*	*Bacteroidales*		
**pH Test Stat**	**pval**	**Phylum**	**Class**	**Order**	**Family**	**Genus**
**-0.6612**	0.0001	*Cyanobacteria*	*Synechococcophycideae*	*Synechococcales*	*Synechococcaceae*	*Synechococcus*

Supplementary Table 3. Kruskal-Wallis enriched taxonomy identified within DCM concentration groups.

Enriched Taxonomy (10,000-100,000 µg/L DCM)	Taxonomy	P
6.608E+02	*Bacteria Blgi18*	2.514E-03
4.745E+02	*YS2*	3.021E-03
2.645E+02	*Ruminococcaceae*	3.133E-03
2.200E+01	*Rikenellaceae Blvii28*	3.103E-03
1.975E+01	*Melioribacteraceae*	1.317E-03
1.750E+01	*Bacteria OD1 ABY1*	1.312E-03
1.175E+01	*Rhodobacteraceae*	1.317E-03
7.500E+00	*Dehalobacterium*	1.317E-03
7.250E+00	*Bacteria OP11*	1.411E-03
3.750E+00	*Deltaproteobacteria NKB15*	1.317E-03
3.000E+00	*Spirochaetes*	1.317E-03
2.250E+00	*Rhodospirillaceae*	1.317E-03
1.750E+00	*Bacteria OD1 ZB2*	1.317E-03
1.750E+00	*Bacteria OD1 ZB2*	1.317E-03
1.250E+00	*Bacteria OD1 ZB2*	1.305E-03
1.250E+00	*Bacteria OD1 ZB2*	1.305E-03
1.250E+00	*Bacteria OP3 PBS-25*	1.305E-03
**Enriched Taxonomy (100,000-1,000,000 µg/L DCM)**	**Taxonomy**	**P**
1.143E+03	*YS2*	3.021E-03
6.667E+00	*Chlamydiae*	2.689E-03
6.333E+00	*Victivallaceae*	3.092E-03
6.000E+00	*GN02 3BR-5F*	9.556E-04
6.000E+00	*K2-4-19*	3.087E-03
4.667E+00	*Candidatus Protochlamydia*	3.092E-03
4.000E+00	*GN02 3BR-5F*	3.092E-03

3.333E+00	*Acidimicrobiales*	3.092E-03
2.333E+00	*Clostridiales*	1.181E-03
1.667E+00	*PRR-11*	3.092E-03
1.000E+00	*TSBW08*	3.092E-03
1.000E+00	*MLE1-12*	3.092E-03
1.000E+00	*Phycisphaerae*	3.092E-03
**Enriched Taxonomy (1,000,000-10,000,000 µg/L DCM)**	**Taxonomy**	**P**
6.500E+00	*Desulfurispora*	5.560E-05
5.750E+00	*Alkalibacterium*	5.560E-05
8.000E+00	*Peptococcaceae*	5.754E-04
4.000E+00	*Desulfotomaculum*	1.305E-03
6.000E+00	*Geobacter*	1.317E-03
3.500E+00	*Sphingobacterium*	1.317E-03
2.750E+00	*Desulfosporosinus meridiei*	1.317E-03
2.750E+00	*Gracilibacteraceae*	1.317E-03
1.250E+01	*Nesterenkonia*	1.681E-03
3.750E+00	*Dehalobacterium*	1.706E-03
2.130E+02	*Elusimicrobiales*	2.653E-03
1.705E+02	*Desulfovibrio aminophilus*	3.647E-03
